# Titanium elastic nails vs locking plate in pediatric subtrochanteric femur fractures: A systematic review and meta-analysis

**DOI:** 10.3389/fped.2023.1114265

**Published:** 2023-03-03

**Authors:** Yaqiang Zhang, Yun Xue, Maosheng Zhao, Xianxia Chen, Qiuming Gao

**Affiliations:** ^1^ Department of Orthopedics, The 940th Hosptial of Joint Logistics Support Force of Chinese People’s Liberation Army, Gansu, China; ^2^ Lanzhou University Second Hospital, Gansu, China

**Keywords:** titanium elastic nail, locking plate, subtrochanteric fractures, pediatric, meta-analyses

## Abstract

**Objective:**

Titanium elastic nails (TENs) and locking plates (LPs) are currently the main internal fixation for treating pediatric subtrochanteric femur fractures, and the optimal choice of internal fixation is controversial. This study aimed to systematically evaluate the effectiveness and safety of TENs and LPs in treating subtrochanteric fractures in children to provide a theoretical basis and reference for clinical treatment.

**Methods:**

The literature related to TENs and LPs for treating subtrochanteric fractures in children was searched using the CNKI, PubMed, Cochrane, Embase, and Web of Science, and the search time frame was from the establishment of the database to October 2022. Two evaluators screened the literature according to the inclusion and exclusion criteria and extracted relevant data. Meta-analysis was performed using Stata14.0 software.

**Results:**

A total of 9 studies with 407 patients with subtrochanteric femur fractures were included in the final screening, including 210 cases with TENs and 197 cases with LPs. Meta-analysis results showed that compared with the locking plate, TEN had a shorter operative time [WMD = −1.3, 95%CI(−1.94,-0.66), *p *< 0.01], less intraoperative bleeding [WMD = −84.45, 95%CI(−111.09, −57.82), *p *< 0.01], shorter fracture healing time [WMD = −1.3, 95%CI(−1.94,−0.66), *P *< 0.01], shorter hospital stays [WMD = −2.80, 95% CI(−4.63,−0.98), *p *< 0.01], and earlier full weight bearing [SMD = −0.48, 95% CI(−0.91,−0.04), *p *< 0.05] but more intraoperative fluoroscopy [WMD = 28.23, 95% CI(15.22,41.25), *p *< 0.05]. The overall complication rate was high [OR = 3.52, 95% CI(1.96,6.34), *p *< 0.05], and the postoperative period was prone to angulation, rotation, and inversion deformity [OR = 3.68, 95% CI(1.40, 9.68), *p* < 0.05]. No significant difference in the incidence of lower limb inequality between the two types of internal fixation [OR = 0.83, 95% CI(0.38, 1.85), *p* > 0.05] and no significant difference in the Harris score of the hip at the last follow-up between the two types of internal fixation [WMD = −0.67, 95% CI(−2.01,0.67), *p* > 0.05] were found.

**Conclusion:**

In comparison to LPs, TENs have a shorter operation time, less intraoperative bleeding, and a shorter fracture healing time, and the child can be fully weight-bearing earlier. Locking plates can reduce the operator's x-ray exposure, and the incidence rate of postoperative angulation, rotation, and inversion deformity is low. Therefore, TENs and LPs are the best internal fixation methods for treating subtrochanteric fractures in children.

## 1. Introduction

Subtrochanteric fractures in children are relatively rare clinically, accounting for only 4%–10% of femur fractures in children (1), mainly due to high-energy injuries from car accidents or high falls. Because of the muscle pull around the femoral rotor in children, the fracture end is easily rotated and displaced deformed, and the proximal end of the fracture is short, which makes the repositioning difficult to achieve and to maintain. Conservative treatment can easily lead to complications such as poor or lost fracture repositioning, hip internal and external rotation deformity, and hip mobility disorders; therefore, surgery is the best choice at present (2). The selection of appropriate internal fixation for subtrochanteric fractures in children is crucial, and the commonly used internal fixation in clinical practice includes elastic intramedullary nails, locked compression plates, and locked intramedullary nails (3). Although the locking intramedullary nail can better control rotation and angulation, it can easily damage the epiphyseal plate and lead to developmental disorders of the limb and is limited by the diameter of the medullary cavity in children, which is not yet accepted by most surgeons (4). For subtrochanteric fractures in children, flexible intramedullary pins and splints are more commonly used for internal fixation, but both choices are still controversial. In our study, we compared the efficacy and safety of flexible intramedullary pins and plates in the treatment of subtrochanteric fractures in children through meta-analysis to find a more suitable internal fixation method for subtrochanteric fractures in children.

## 2. Materials and methods

This study was reported in line with the 2020 Preferred Reporting Items for Systematic Reviews and Meta-Analyses (PRISMA) and Assessing the Methodological Quality of Systematic Reviews (AMSTAR) guidelines (5, 6).

### 2.1. Materials

#### 2.1.1. Literature search

In Cochrane, PubMed, Web of Science, Embase, and the Chinese database China National Knowledge Infrastructure, the search time was from the creation of the database to September 2022, and the search terms were “subtrochanteric femur fractures, elastic stable intramedullary nail or titanium elastic nail, and locking plate.”

#### 2.1.2. Inclusion and exclusion criteria

The inclusion criteria are as follows:

Population: patients with subtrochanteric femur fractures;
Intervention: patients treated with titanium elastic nails (TENs);
Comparator: patients treated with locking plates;
Outcomes: operative time, intraoperative bleeding, fracture healing time, length of hospital stay, time to full weight bearing, final hip score, number of intraoperative fluoroscopies, angular rotational deformity, lower limb inequality, and incisional infection, Including at least two above outcomes in study.
Study design: retrospective and prospective comparative studies.
The exclusion criteria are as follows: (1) age <5 years, >15 years; (2) pathological fractures; (3) biomechanical or cadaveric studies; and (4) reviews, conference abstracts, expert opinions, review articles, meta-analyses, and case reports.

#### 2.1.3. Statistical extraction

Two evaluators independently screened the relevant literature according to the search strategy and inclusion and exclusion criteria and then cross-checked the literature. In instances of disagreement, the two persons discussed and decided, or the third evaluator intervened if necessary. Data extraction included the following: (1) basic characteristics of the study, including the authors' names, year of publication, study design, sample size, age, gender, Seinsheimer classification, and follow-up time; and (2) outcome indicators, including operative time, intraoperative bleeding, fracture healing time, hospital stay, number of fully weight-bearing intraoperative fluoroscopies, angular rotational deformity, lower extremity inequality, time, final hip score, overall complications.

#### 2.1.4. Evaluation of the quality of the included literature

Two evaluators independently evaluated the included studies, and randomized controlled trials (RCTs) were assessed for quality using the Newcastle–Ottawa Scale (NOS) according to the type of study included, with a score of more than 6 indicating high-quality literature. Nonrandomized studies were evaluated using the Research Evaluation Instrument of Non-Randomized Studies – of Interventions (ROBINS-I), and the final results were scored according to the correlation with the total risk of bias (low, moderate, high, very high, and uninformative) (7).

### 2.3. Statistical analysis

Stata version 14.0 was used for data analysis, weighted mean difference (WMD) was used as the effect size for continuous variables, standardized mean difference (SMD) was used as the effect size for different units, and odds ratio (OR) was used as the effect size for dichotomous variables. When *p *< 0.1 or *I*^2^ > 50% were considered to have significant heterogeneity, a random-effects model was used for statistical analysis; *p *> 0.1 or *I*^2^ < 50% were considered to have less heterogeneity, and a fixed-effects model was used for statistical analysis. Sensitivity analysis was performed to confirm the stability of the study results, and Egger's test was used to analyze publication bias when ≥5 studies were included, with a test level of *α* = 0.05.

## 3. Results

### 3.1. Literature search results

**Figure 1:**
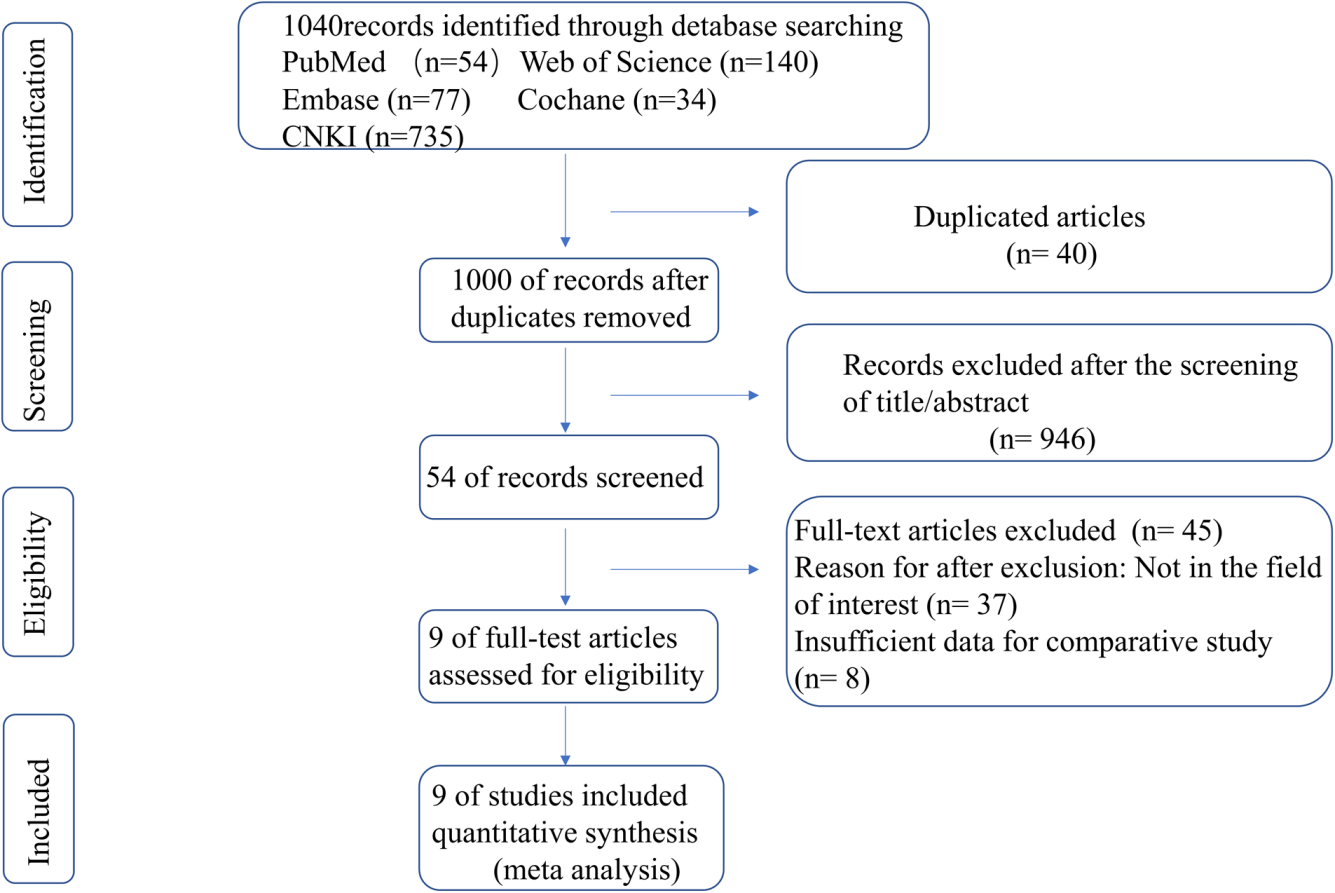
Flow diagram of study selection; *n* = number of studies.

A total of 407 articles were obtained from the preliminary search. After eliminating duplicate articles, articles that did not meet the inclusion criteria, and those for which data could not be extracted, a total of 9 articles (8–16) were included in this study (Figure 1). The included literature was a retrospective cohort study with a total of 407 children with a subtrochanteric femur, including 210 cases in the TEN group and 197 cases in the LP group. The general characteristics of each study are presented in Table 1.

**Table 1:** Baseline characteristics of studies.

Author	Study design	Cases (*n*)		Mean age (years)		Gender (M/F)		Seinsheimer classification (LP/TEN)					Follow-up (months)
		LP	TEN	LP	TEN	LP	TEN	I	II	III	IV	V	
Fang et al. (2012)	RCS	25	17	10.5		29/13		1	12	22	7	0	24–72
Zhu et al. (2013)	RCS	18	18	9.6 ± 1.30	9.4 ± 1.90	10/8	11/7	0/0	10/9	6/7	2/2	0/0	15–36
Li et al. (2013)	RCS	29	25	8.4	7.9	21/8	21/4	NA	NA	NA	NA	NA	>4
Sun et al. (2015)	RCS	26	26	12.2 ± 2.5	11.6 ± 2.0	11/15	9/17	0/0	20/18	6/8	0/0	0/0	23–48
Xu et al. (2017)	RCS	16	19	10.7	9.8	9/7	11/8	0/0	9/11	5/7	2/1	0/0	6–24
Xu et al. (2018)	RCS	30	37	9.4	6.8	18/12	22/15	NA	NA	NA	NA	NA	16–42
Sun et al. (2020)	RCS	22	34	8.23 ± 1.27	6.65 ± 1.48	14/8	22/12	NA	NA	NA	NA	NA	13–48
Li et al. (2022)	RCS	15	18	9.33 ± 2.01	8.58 ± 1.76	17/11	11/7	0/0	8/11	4/6	3/1	0/0	>18
Hong et al. (2022)	RCS	16	16	8.4 ± 1.4	8.4 ± 1.5	7/9	7/9	2/2	4/4	10/10	0/0	0/0	>24

*n*, number of studies; LP, locking plate; TEN, titanium elastic nail; M, male; F, female; NA, not available.

### 3.2. Quality evaluation

As the included studies were retrospective cohort studies, the ROBINS-I evaluation tool was used to evaluate the quality of the nine included studies: five low-risk items, three moderate-risk items, and one high-risk item (Table 2).

**Table 2:** Quality assessment using the ROBINS-I tool.

Study	Confounding	Selection	Intervention classification	Deviation from intervention	Missing data	Measurement of outcome	Selection of reported result	Overall
Fang et al. (2012)	Low	Low	Moderate	Low	Moderate	Low	Low	Moderate
Zhu et al. (2013)	Low	Moderate	Moderate	Low	Moderate	Low	Low	Serious
Li et al. (2013)	Low	Low	Moderate	Low	Low	Low	Low	Moderate
Sun et al. (2015)	Low	Moderate	Moderate	Low	Low	Low	Low	Moderate
Xu et al. (2017)	Low	Low	Low	Low	Low	Low	Low	Low
Xu et al. (2018)	Low	Moderate	Low	Low	Low	Low	Low	Low
Sun et al. (2020)	Low	Low	Moderate	Low	Low	Low	Low	Low
Li et al. (2022)	Low	Low	Low	Low	Low	Low	Low	Low
Hong et al. (2022)	Low	Low	Low	Low	Low	Low	Low	Low

### 3.3. Results of a meta-analysis

#### 3.3.1. Operative time

**Figure 2:**
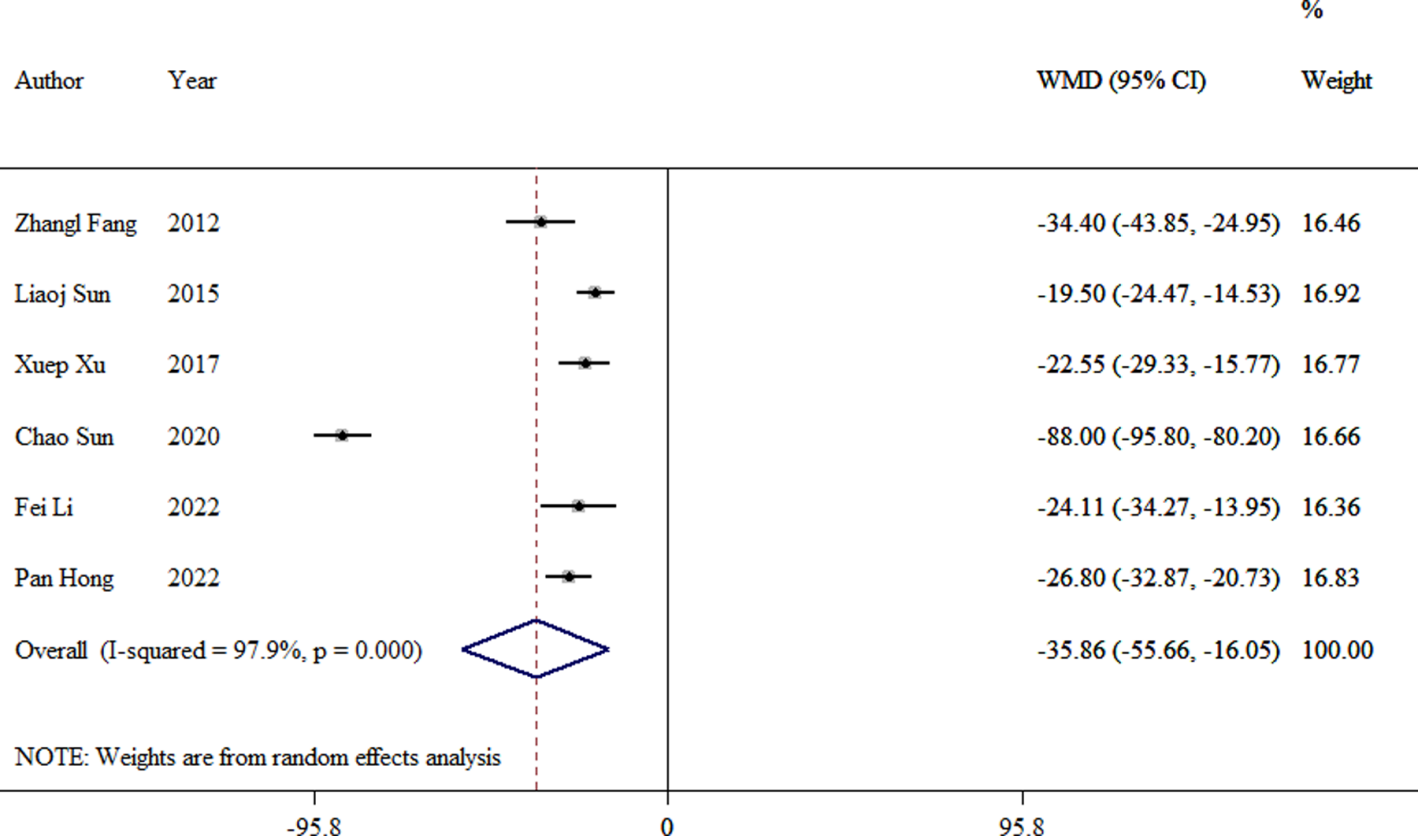
Forest plot of the meta-analysis of operative time.

Data were extracted from six studies to evaluate the operation time (8, 10, 12–14, 16). There was a significant difference between the TEN and LP groups (WMD = −35.86, 95% CI: −55.66–16.05, *p *< 0.01; Figure 2).

#### 3.3.2. Intraoperative bleeding

**Figure 3:**
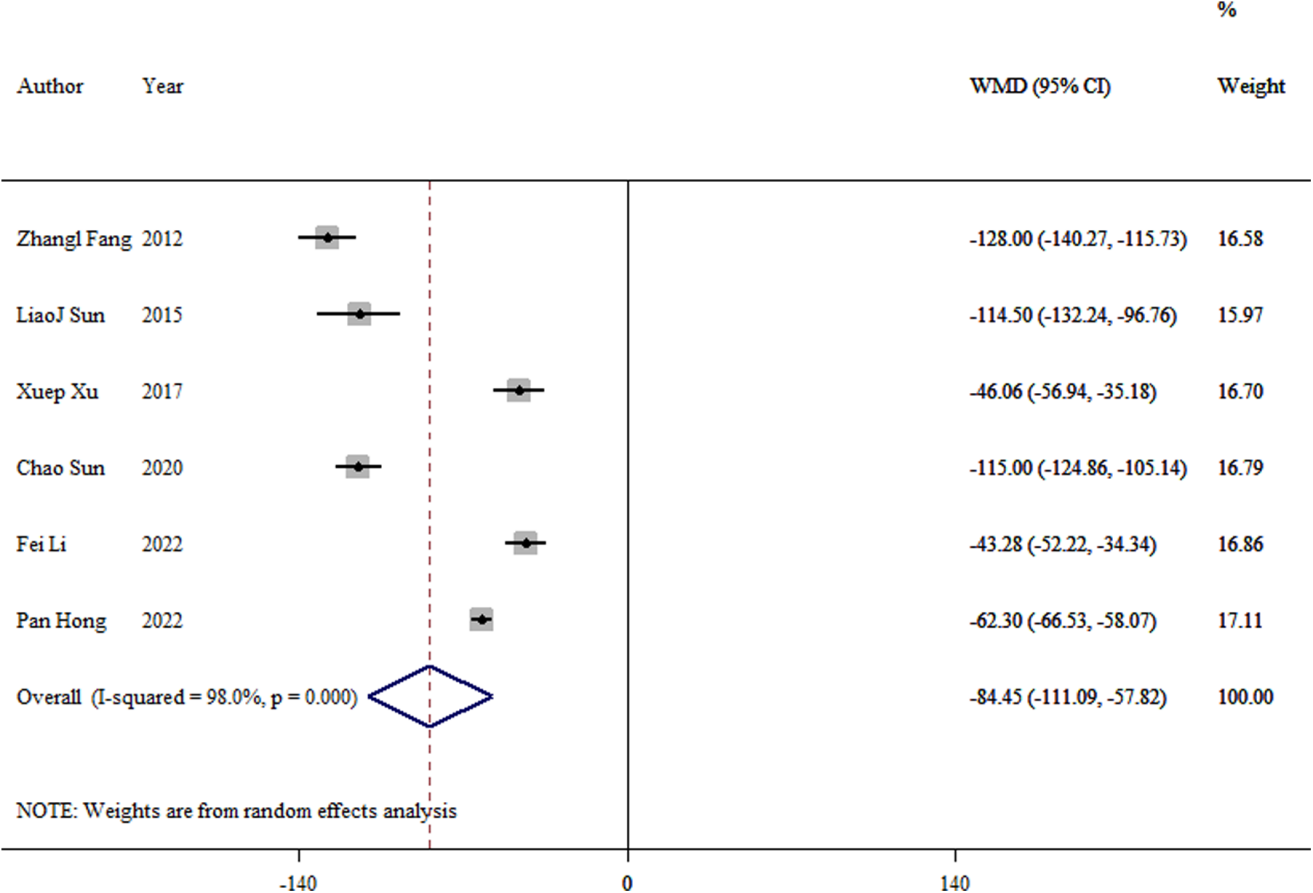
Forest plot of the meta-analysis of intraoperative bleeding.

Data from six studies reported blood loss during the operation (8, 10, 12–14, 16). A significant difference was found between the TEN and LP groups (WMD = −84.45, 95% CI: −111.09–57.82; *p *< 0.01; Figure 3).

#### 3.3.3. Frequency of intraoperative fluoroscopy

**Figure 4:**
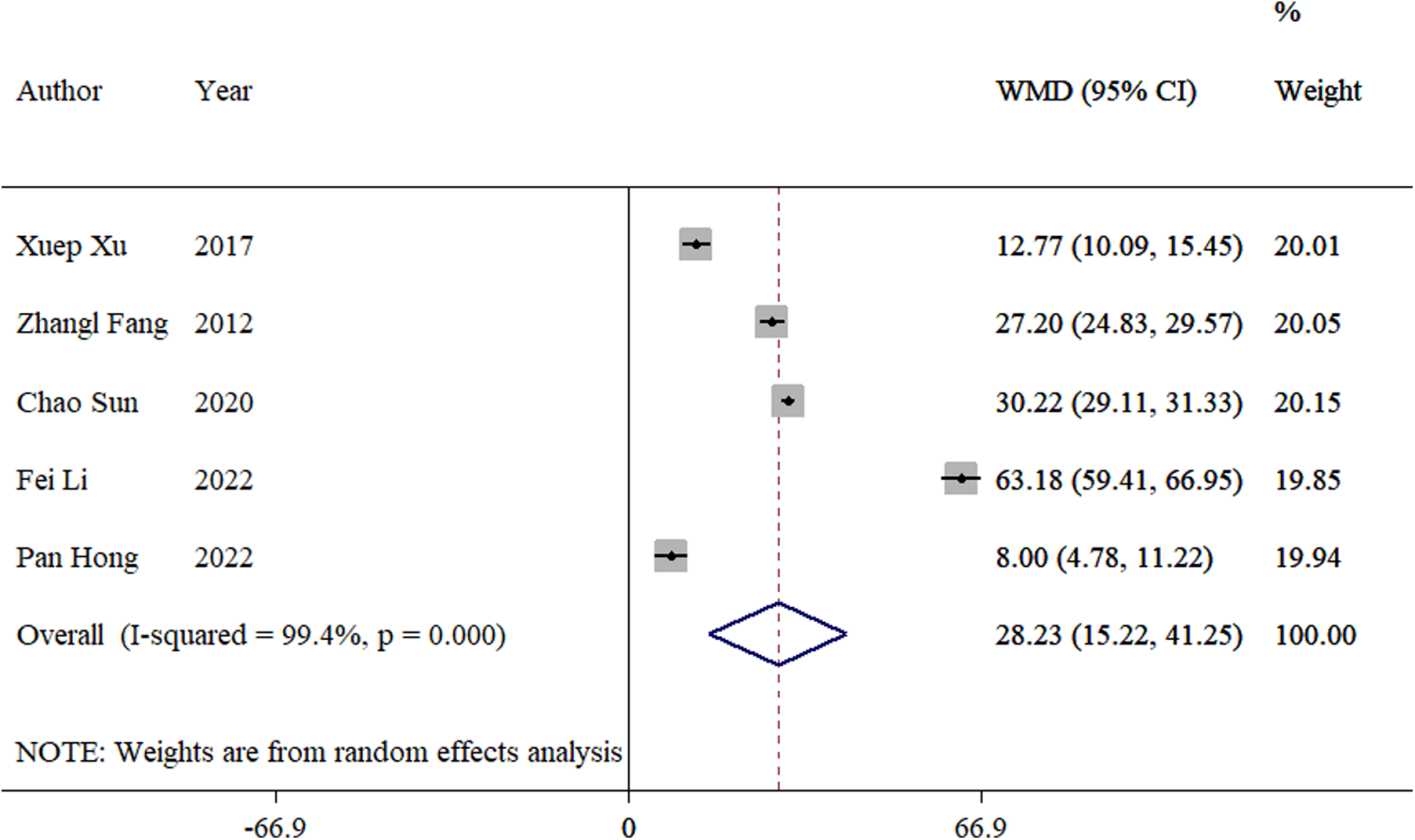
Forest plot of the meta-analysis of the frequency of intraoperative fluoroscopy.

The frequency of intraoperative fluoroscopy was reported in five studies (8, 12–14, 16). The meta-analysis showed a significant difference between the TEN and LP groups (WMD = 28.23, 95% CI: 15.22–42.25; *p *< 0.01; Figure 4).

#### 3.3.4. Hospital stay

**Figure 5:**
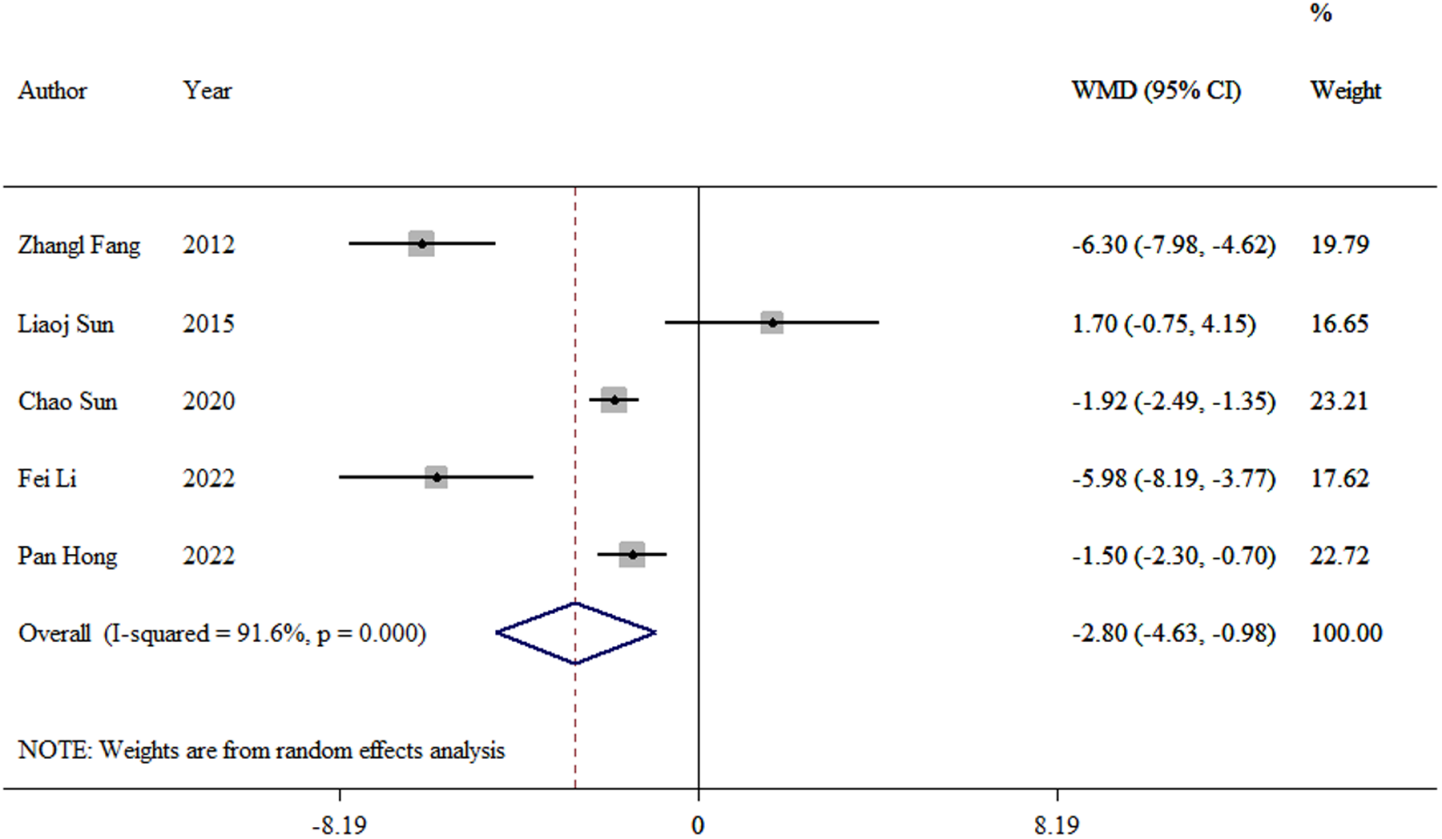
Forest plot of the meta-analysis of hospital stay.

We extracted the length of hospital stay data from five studies (8, 10, 12–14). The meta-analysis revealed a significant difference between the TEN and LP groups (WMD = −2.8, 95% CI: −4.63–0.99; *p* = 0.03; Figure 5).

#### 3.3.5. Fracture healing time

**Figure 6:**
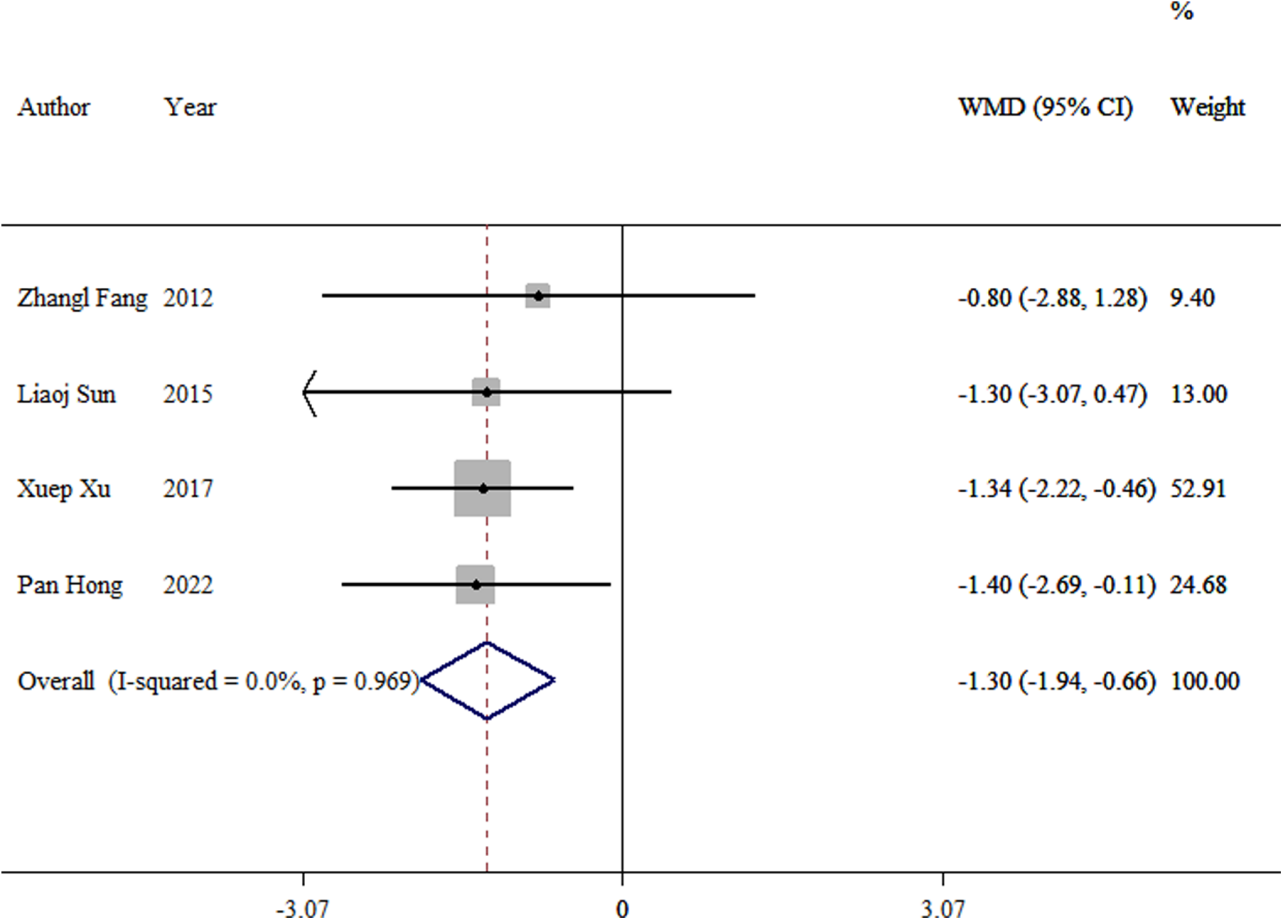
Forest plot of the meta-analysis of fracture healing time.

Four studies (8, 10, 13, 16) were incorporated into the analysis. The meta-analysis showed a significant difference between the TEN and LP groups (WMD = −1.30, 95% CI: −1.94–0.66; *p *< 0.01; Figure 6).

#### 3.3.6. Time to full weight bearing

**Figure 7:**
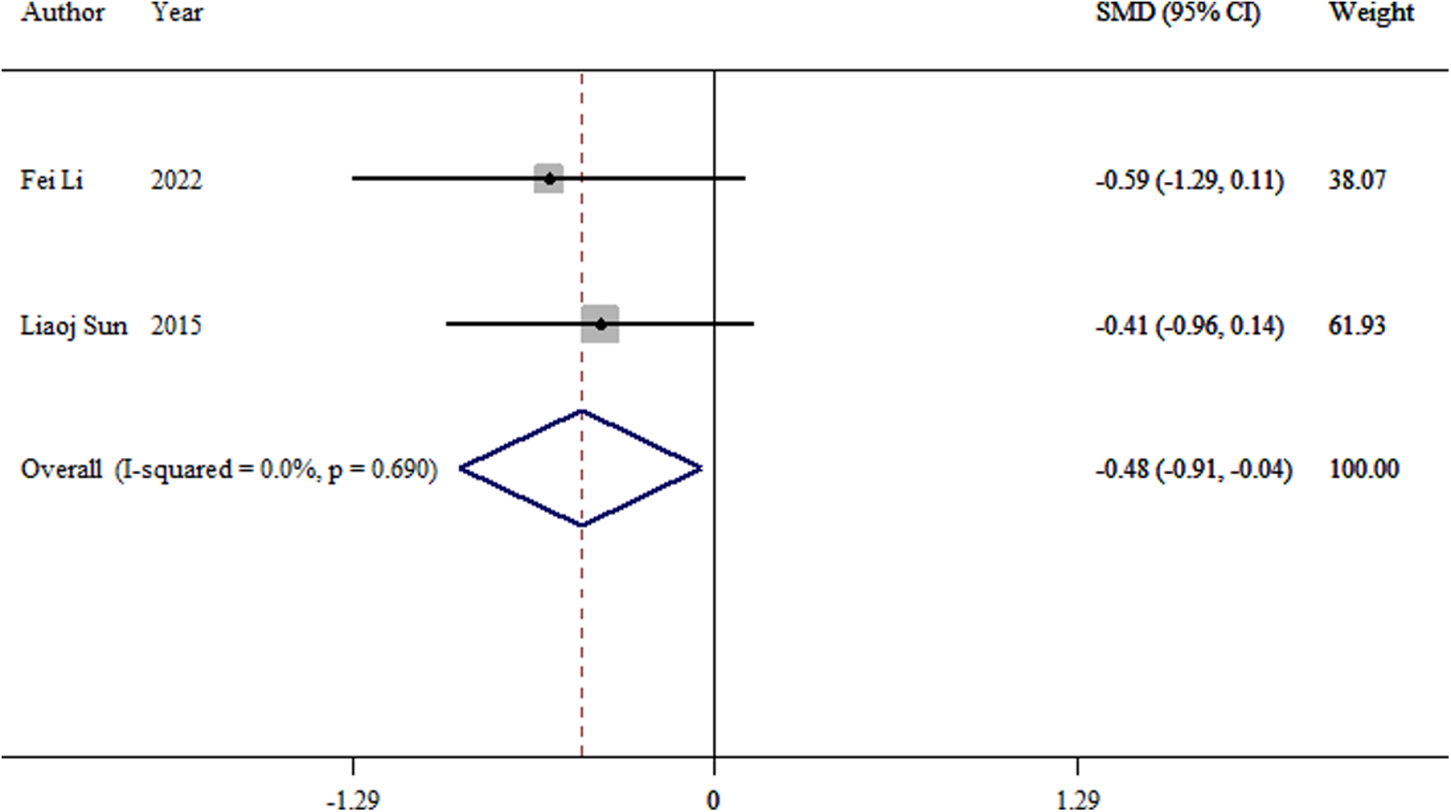
Forest plot of the meta-analysis of time to full weight bearing.

Two studies (10, 14) analyzed the time to full weight bearing after surgery between the two groups. The meta-analysis revealed a significant difference between the TEN and LP groups (SMD = −0.48, 95% CI: −0.91–0.04; *p *= 0.03; Figure 7).

#### 3.3.7. Hip Harris scores

**Figure 8:**
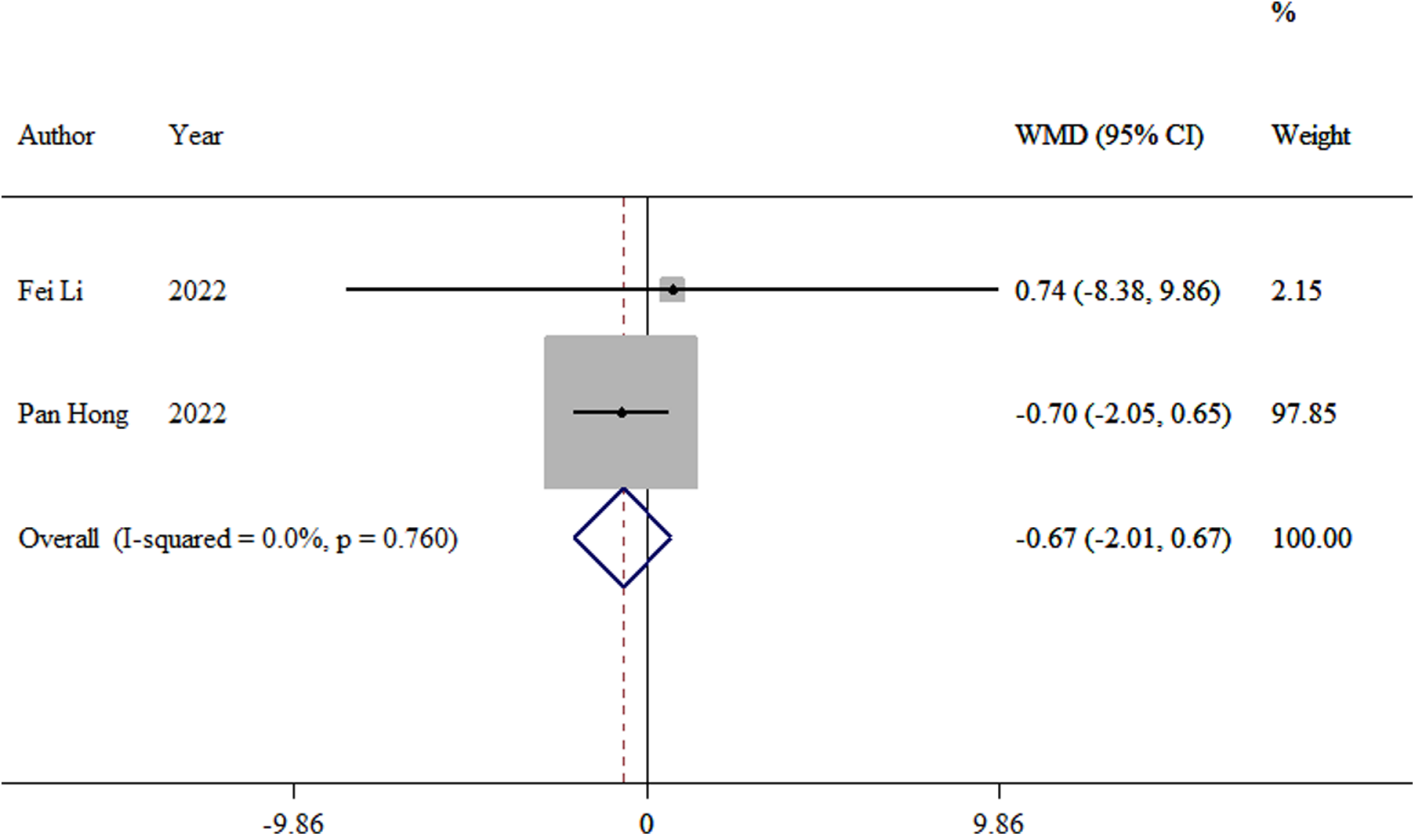
Forest plot of the meta-analysis of hip Harris scores.

Two studies (13, 14) compared the postoperative hip Harris scores between the two groups. The meta-analysis showed no significant difference in Hip Harris scores between the TEN and LP groups (WMD = −0.67, 95% CI: −2.01–0.67; *p *= 0.327; Figure 8).

#### 3.3.8. Overall complication

**Figure 9:**
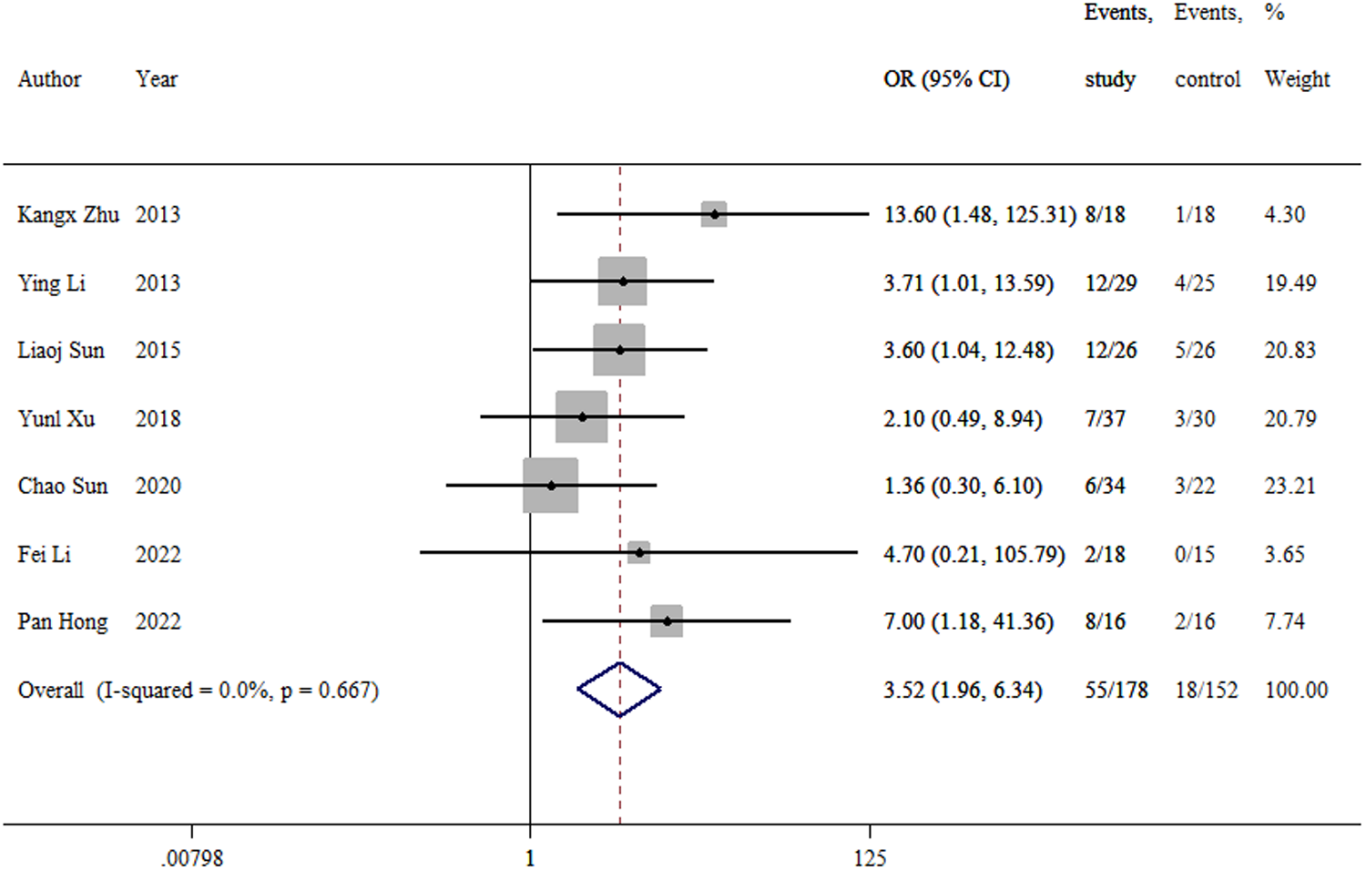
Forest plot of the meta-analysis of overall complications.

The overall complication was reported in seven studies (9–15). No difference was observed in the rate of overall complications between the two groups (OR = 3.52, 95% CI: 1.96–6.34; *p *= 0.058; (Figure 9). The overall complication rate was 30.9% in the TEN group and 11.8% in the LP group.

#### 3.3.9. Major complications

**Figure 10:**
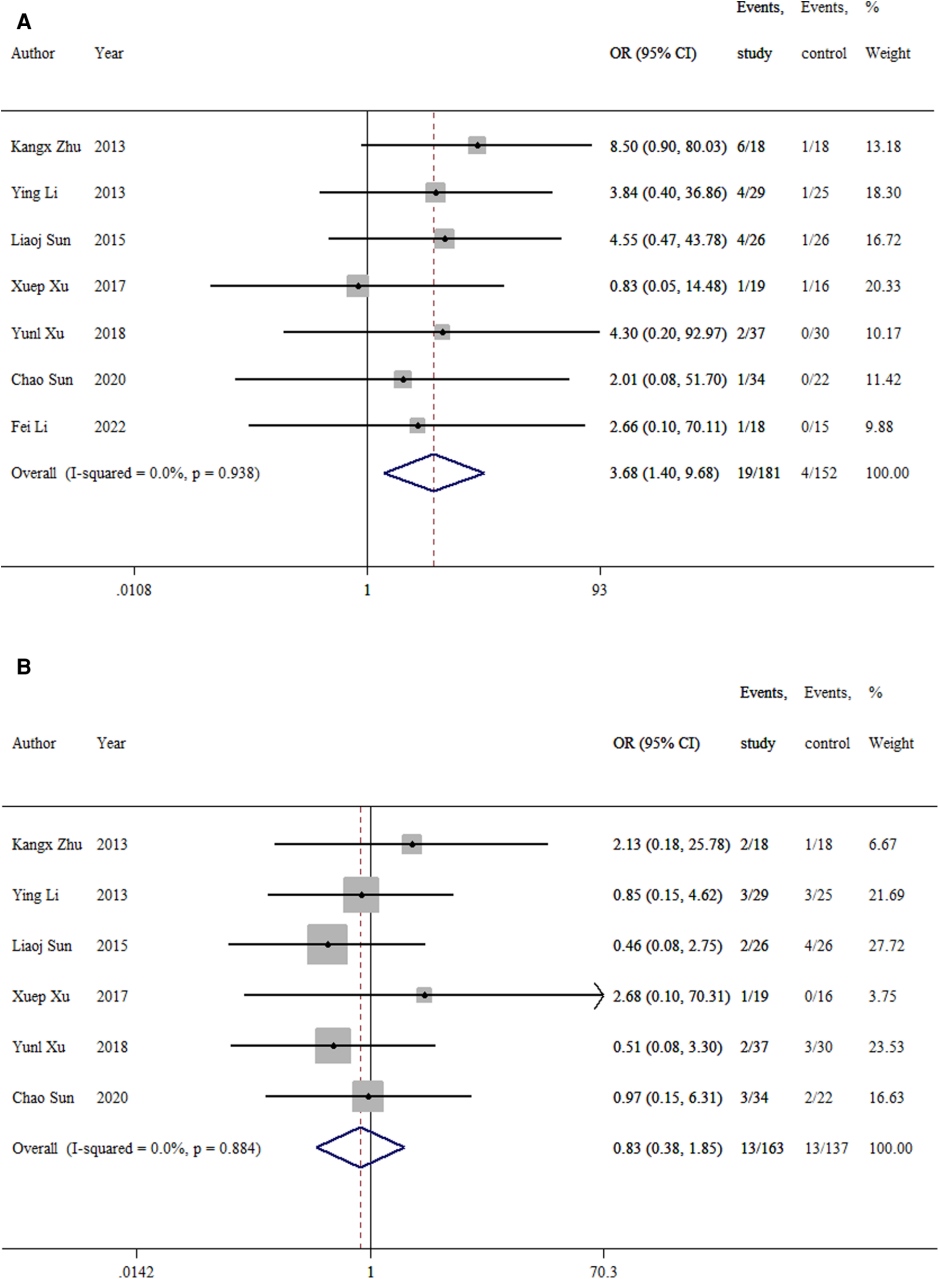
Forest plot of the meta-analysis of major complications. (**A)** rotational, angular, and valgus deformity; (**B)** lower extremity inequality.

Seven studies (9–12, 14–16) reported postoperative rotational angular deformity. The meta-analysis showed a significant difference between the TEN and LP groups (OR = 3.68, 95% CI: 1.40–9.68; *p *= 0.008; Figure 10A). The incidence was 10.5% in the TEN group and 2.6% in the LP group.

Six studies (9–12, 15, 16) reported lower extremity inequality. No difference was found in the rate of lower extremity inequality between the two groups (OR = 0.84 (95% CI: 0.38–1.85; *p *= 0.66; Figure 10B). The incidence was 8.0% in the TEN group and 9.5% in the LP group.

### 3.4. Publication bias and sensitivity analysis

Egger's regression test for publication bias was performed on the included literature and the results. The results suggest no significant publication bias, and the selected studies were well-represented (Supplementary Table S1). Sensitivity analysis was performed on the combined effect sizes for each outcome indicator, and the results after removing each of the studies, one by one, did not change significantly from the results before removal, demonstrating that the combined results were stable (Supplementary Figure S1).

## 4. Discussion

A subtrochanteric fracture in children is defined as a fracture in which the ratio of the length of the lower edge of the lesser trochanter from the highest point of the fracture to the total length of the femur is less than 10% (17). Because the subtrochanteric femur in children is the area where the proximal femoral epiphysis migrates toward the femoral stem, it is the main concentration point of stress, and there are differences in bone shaping ability compared to the femoral stem, as well as the role of gluteus and iliopsoas muscle pulling after a subtrochanteric fracture. The fracture end is unstable and prone to angulation and shortening deformity. Because of the characteristics of subtrochanteric fractures in children, conservative treatment is difficult to reset and it is also difficult to maintain the reset, and surgical treatment can achieve satisfactory clinical results compared with conservative treatment. Due to the special characteristics of intertrochanteric fractures in children, the selection of internal fixation methods for surgical treatment is still very controversial. TEN and locking plates are the main internal fixation methods for treating subtrochanteric fractures in children, but both of them have certain shortcomings, and understanding their shortcomings can facilitate the better selection of the internal fixation method. In this study, we found that compared with locking plates, TENs have a shorter operation time, less intraoperative bleeding, a shorter length of hospital stay, and a shorter fracture healing time, and the child can weight-bear earlier, but intraoperative fluoroscopy is needed repeatedly to determine the fracture end repositioning and TEN positions, which increases the operator's x-ray exposure. Also, the postoperative nail tail irritates the skin and causes pain and discomfort to the child. The bone plate can be strongly fixed internally, which reduces the occurrence of fracture end displacement and deformity healing, but the intraoperative bleeding and operation time are long, and the blood supply to the fracture end is destroyed, which affects the fracture healing.

The choice of internal fixation for subtrochanteric fractures in children depends on the type of fracture, weight, and age. Milligan et al (18) and Shah et al (19) concluded that treating subtrochanteric fractures in children, especially in overweight children with comminuted, or long oblique unstable fractures, is superior to elastic nail fixation and that internal fixation with a splint can provide strong internal fixation and achieve anatomic repositioning. Because the subtrochanteric fracture in children is located at the proximal end of the femur, the support point of the elastic intramedullary pin is far away from the fracture end and the proximal fixation space is limited, which is not favorable to maintaining the stability of the fracture end and has a higher incidence of postoperative rotation, angulation, and inversion deformity complications (20). Bone splints can reduce complications caused by destabilization of the TEN. This is consistent with the results of our study, and the results of this meta-analysis also revealed a higher incidence of postoperative rotation, angulation, and valgus deformities with TENs than with locking plates. Because children are in the growth and development stage and have strong bone-shaping abilities, they can self-correct later and ultimately experience less of an impact on hip function. This study also confirmed no significant difference in the Harris score at the end hip, indicating better recovery of hip function after both types of internal fixation. However, Basa et al. (21) found satisfactory clinical results with TEN for any type of subtrochanteric fracture in children without severe rotational or inversion deformities. The study by Alberghina et al. (22) also concluded that displaced subtrochanteric fractures of the femur under 14 years of age achieved good results with TEN, but the studies by Basa et al. (21) and Alberghina et al. (22) lacked a controlled comparison with locking plates and could not show that TENs are superior to splints in the treatment of comminuted and unstable subtrochanteric fractures in children.

Based on the disadvantages of TEN, some scholars have improved the internal fixation method. Conventional TEN internal fixation is the lateral TEN crossing the fracture end and entering under the greater trochanteric epiphysis and the medial TEN to the level of the femoral spur or femoral neck to avoid damage to the femoral epiphysis. Cha et al. (23) improved TEN methods to increase the stability of TEN fixations by penetrating the apophysis of the greater trochanter and the medial TEN through the posterior neck cortex in all patients. After at least 5 years of follow-up, there was no difference in hip function compared to the healthy side, and no angular deformity or severe inequality of the lower extremity (<2 cm) was found. Hong et al. (13) achieved satisfactory clinical results in their study with no significant angular deformity by fixation with three elastic nails to obtain stability of the fracture end. Memeo et al. (24) and Kayaokay et al. (25) concluded that elastic intramedullary pins are suitable for patients weighing less than 50 kg and in children aged 6–15 years. Although Cha et al. and Sun et al. achieved some results, their study on children weighing less than 50 kg does not suggest that the modified TEN approach is suitable for children above 50 kg and needs further investigation. Although the weight of the children was not specified in the literature included in this study, the age of the included children was 5–15 years old. It was found that the overall complication rate after TEN was high, and the incidence of angulation, rotation, and internal rotation (10.5%) was higher than that of locking plates (2.6%) because children's bones are more plastic than adult bones. The follow-up time of this study was short, and angulation and internal rotation deformities may be improved after long-term follow-up. The study was not based on fracture stability. Also, this study was not grouped according to fracture stability, so it cannot be stated whether TEN is suitable for unstable fractures. However, based on the low trauma of the TEN, short fracture healing time, early weight-bearing of the child, and ease of removal of the internal fixation, the authors concluded that TEN could be selected for internal fixation in children of this age and weight.

Internal fixation with locking plates may not provide a sufficient number of fixation points on the proximal segment due to the small distance between the fracture and the epiphyseal plate, and only 1–2 screws can be used proximally at most, which may result in reduced fracture stability and the inability of the child perform early weight-bearing exercises (26). Danisman et al. (27) and Gogna et al. (28) suggested that an adult proximal humeral locking plate (3.5 mm) may be a good option for treating subtrochanteric fractures in children, with its easy fit to the child's proximal femur, its wide proximal shape enhancing the grip on the proximal femur, multiple screw holes in the proximal portion of the plate allowing multiple locking screws to be inserted into the child's femoral neck in multiple directions, resulting in greater angular stability, and its satisfactory results in the clinical application without lower extremity inequalities. Although this study cannot explain the superiority of the splint over the flexible intramedullary pin in unstable subtrochanteric fractures, based on the special nature of the subtrochanteric femur in children and the inability of the flexible intramedullary pin to better control the axial displacement and fracture end rotation, the authors believe that internal fixation with the splint may be considered in children with unstable subtrochanteric femur fractures weighing more than 50 kg, and further studies are needed on the selection of the locking plates.

The limitation of this study is that the included articles are retrospective cohort studies, which are prone to selective bias. Heterogeneity in the included studies is unavoidable because the procedures were performed by different surgeons, as well as because of gender, age, and ethnicity differences in the patients. The small sample size also presents some limitations. Therefore, this study needs to be further validated by a prospective randomized controlled trial with a large sample size. Also, there was considerable interstudy heterogeneity in this study, which may be due to the effects of age and body weight in each study.

## 5. Conclusion

Compared with locking plates, TENs have a shorter operative time, less intraoperative bleeding, a shorter length of hospital stay, and a shorter fracture healing time, and the child can weight-bear earlier. At the same time, children recover well regarding hip function after TENs and locking plates; therefore, TENs remain a viable option for school-aged children with subtrochanteric fractures.

## Data Availability

The original contributions presented in the study are included in the article/**Supplementary Material**, further inquiries can be directed to the corresponding author.
